# Development of Cardiorespiratory Fitness in Children in the Transition From Kindergarten to Basic School According to Participation in Organized Sports

**DOI:** 10.3389/fphys.2022.881364

**Published:** 2022-06-27

**Authors:** Merike Järvamägi, Eva-Maria Riso, Kirkke Reisberg, Jaak Jürimäe

**Affiliations:** Faculty of Medicine, Institute of Sports Sciences and Physiotherapy, University of Tartu, Tartu, Estonia

**Keywords:** cardiorespiratory fitness, organized sports, body composition, physical activity, children

## Abstract

**Purpose:** This study examined the development of cardiorespiratory fitness (CRF) in children in the transition from kindergarten to basic school according to participation in organized sports and estimated the associations of CRF and body composition indices during the transition from childhood to preadolescence.

**Methods:** Children participated in the three-staged study (kindergarten, 6.6 years, *n* = 212; 1st grade, 7.6 years, *n* = 136; and 5th grade, 11.5 years, *n* = 142) for 5 years and were categorized into three groups according to their participation in organized sports in the study period (whole period, episodically, and never). Cardiorespiratory fitness was assessed by performing a 20-m shuttle run test, while body composition was measured by skinfold thicknesses, and physical activity was registered with an accelerometer. International gender- and age-specific CRF reference normativities were also used to characterize the participants.

**Results:** Children who participated consistently in organized sports had significantly higher CRF levels and lower body fatness (31.3 ± 13.5 laps in 20 m shuttle run; 21.1% ± 6.3% body fat) in 11.5 years than in children who had never participated in sports clubs (20.7 ± 12.0 laps in 20-m shuttle run; 26.1% ± 6.8% body fat). Body composition and CRF did not associate in consistently trained children. The proportion of 5th grade children demonstrating age-appropriate healthy CRF was almost threefold higher in the group of consistent sports training among both boys and girls than among non-members of sports clubs.

**Conclusion:** Consistent attendance in organized sports in childhood and early preadolescence ensures higher CRF and healthier body composition than in children who had no experience of organized sports.

## Introduction

Public health recommendations suggest that 5–17-year-old children and youth should perform at least 60 min of moderate-to-vigorous-intensity physical activity (MVPA) daily (WHO, 2020). Objectively measured physical activity (PA) in European children showed that 43.1% met the suggested daily PA recommendations (Cristi-Montero et al., 2019), while globally, only around 19% of 11–17-year-old children met the daily PA recommendation (Guthold et al., 2020). In Estonia, 11% of 7–9-year-old children (Riso et al., 2016) and 4.3% of 10–12-year-old children (Riso et al., 2018) were sufficiently active as estimated according to WHO PA recommendations (WHO, 2020). Physical activity and sedentary behavior in youth vary by country, age, and sex, but it has been found that the older the children get, the greater time is spent sedentary (Cooper et al., 2015). At present, there is not enough PA to have a positive impact on body composition and CRF parameters in children during growth and maturation. Previous studies have confirmed that participation in organized youth sports in primary school and continuing through adolescence appears to increase the likelihood of a physically active lifestyle in young adulthood (Telama et al., 2006). Several studies indicate that participation in organized sports seems especially important in relation to high PA levels which are associated with higher levels of CRF (Graham et al., 2011; Marques et al., 2016). The lack of more active PA necessary for normal development and health of children leads to the importance of organized sports that could provide active time for children.

Cardiorespiratory fitness and PA are positively associated in children during growth and maturation (Kristensen et al., 2010). As critical physiological changes take place during childhood and preadolescence, engagement in various types of PA has numerous health benefits, including an increase in CRF (Vicente-Rodríguez, 2006). A recent study shows that higher levels of pubertal CRF, and not PA, were associated with lower body fatness indices in late adolescence (Remmel et al., 2021). Longitudinal studies have demonstrated that increased PA is protective against relative fatness and weight gains during growth and maturation (Must and Tybor, 2005). Physical activity in childhood and adolescence predicts CRF in adulthood (Tomkinson et al., 2016). Increased PA does not always reflect in weight loss because fat mass (FM) loss could be compensated by an increase in fat-free mass (FFM) (Westerterp et al., 1992). Although exercise training may not have a long-term effect on body weight loss, it can result in healthier body composition (Westerterp, 2018). Physical activity levels and CRF have been shown to be associated with lower body fatness among children and adolescents (Lätt et al., 2015; Riso et al., 2019b). However, the associations between PA and CRF are not so clear. Accordingly, it is important to understand the possible associations between PA and CRF during growth and maturation.

Youth sports training sessions should contain exercises for bone development, muscular fitness, speed, and agility (Vicente-Rodríguez, 2006). It has been suggested that high-intensity training and increasing the time spent in vigorous PA (VPA) should be a major goal in present and future public health promotion policies, and PA programs should be designed to improve CRF (Ortega et al., 2007). Associations between PA and body composition indices have been found in children, while these results highlight the importance to include VPA in the everyday routine (Reisberg et al., 2020). Accordingly, it is necessary to study the relations of consistent training with body composition indices during the growth and maturation of children.

The purpose of this study was to assess the development of CRF in children over a 5-year period in the transition from kindergarten to basic school according to participation in organized sports. The secondary aim of the study was to estimate the associations of CRF with body composition indices during the transition from childhood to preadolescence.

## Materials and Methods

### Participants

A three-staged study was carried out, evaluating the development of CRF and its associations with body composition indices and PA in the last year in kindergarten, 1st grade, and 5th grade at school. The last year in kindergarten and first year at school were chosen as the measurement points to evaluate the changes in habits of PA after the transition from kindergarten to school, which is a great change in a child’s life and daily schedule. The third measurement in 5th grade reflects the transition from elementary school to basic school, with more school lessons and sedentary time accompanied by reaching a pre-pubertal age. In the first stage of the study, the 6–7-year-old children from 13 randomly selected kindergartens in the city of Tartu and surrounding communities were invited to participate. The parents or caregivers of 400 families were provided with written information about the study, and 284 families agreed to take part in the study. The study procedures were carried out from March to May 2016 (Riso et al., 2019a; Riso et al., 2019b), and 256 6–7-year-old children took part in the measurements. One year later, the same cohort was asked to participate in the second stage of the study. The children were 7–8 years old and studied at the 1st grade in the school. The consent to participate was given by 200 families, and the final sample of the second study stage consisted of 147 children (Reisberg et al., 2021). The same parents and children who had participated in the first and second stages of the study were re-invited to participate in the third stage of the study. The children were 11–12 years old and studied at the 5th grade in school. All families who had participated in the previous stages of the study, including those who missed the second stage, were invited to participate. From 200 agreed families, 143 children provided data for the third stage of the study. The final sample for data analysis in the three-step study was formed from 212 (110 boys and 102 girls) children in the first stage, 136 (70 boys and 66 girls) children in the second stage, and 143 (72 boys and 71 girls) children in the third stage of the study. The study was approved by the Medical Ethics Committee of the University of Tartu, Tartu, Estonia (references 254/T-16; 266/T-8; 299/T-23).

### Assessment of Physical Activity

The hip-worn triaxial Actigraph GT3X accelerometer (ActiGraph LLC, Pensacola, FL, United States) was used to measure PA and sedentary behavior objectively (Riso et al., 2016; Riso et al., 2018). Children were asked to wear the accelerometer for seven consecutive days during their wake-up time, except for during water-based activities. Only the data from children who completed at least 3 days, including one weekend day, with at least 10 h of accelerometer wear time, were valid for further analysis (Riso et al., 2016, 2018). The accelerometers were set on 15-s epochs. The night-time periods when the unit was removed and all sequences of 20 min or more of consecutive zero counts were excluded from the analysis. Readings less than 100 counts per min were treated as sedentary behavior. Counts of 100–2,295, 2,296–4,011, and ≥4,012 per min distinguished light PA (LPA), moderate PA (MPA), and VPA, respectively (Evenson et al., 2008). Time spent in MVPA was calculated as the sum of MPA and VPA.

### Assessment of Body Composition

Height and body mass were measured using standardized procedures as previously described (Riso et al., 2016; Riso et al., 2018). The body mass index (BMI) was calculated as body mass (kg) divided by body height squared (m^2^). Triceps and subscapular thicknesses were measured in triplicate on the right side of the body with a Holtain caliper (Crymmych, United Kingdom) to the nearest 0.2 mm using standardized procedures (Marfell-Jones, 2006). The percentage of body fat (body fat %) and fat mass (FM, kg) was calculated from triceps and subscapular skinfold thicknesses (Slaughter et al., 1988). Fat-free mass (FFM, kg) was derived by subtracting FM from total body mass. In addition, the FFM index was calculated (FFMI; kg.m^−2^).

### Assessment of Cardiorespiratory Fitness

Cardiorespiratory fitness of the children was evaluated by performing the 20-m shuttle run test from a standardized PREFIT test battery (Cadenaz-Sanchez et al., 2016a) that is similar to the tests of the ALPHA test battery and allows to use them in longitudinal studies (Ortega et al., 2015). The running pace was set by the audio signals that set the speed at different stages. The test started at a pace of 8.5 km per hour and increased by 0.5 km per hour every minute. The number of laps was recorded when scoring this test (Ortega et al., 2015). International normative 20-m shuttle run values from 1,142,026 children and youth representing 50 countries were used to evaluate the compliance to age-appropriate normatives of CRF (Tomkinson et al., 2016).

### Sports Club Participation

The parents were asked about the sports club participation of their children in every stage of the study. The children were categorized retrospectively, after the third measurement in the 5th grade, according to their participation in organized sports during all study stages. The members of sports clubs during the whole study period formed group 1; the children who had participated in sports clubs at least in the third stage of the study or second and third stages formed group 2; and the children who had not participated in any organized sports clubs formed group 3.

### Statistical Analysis

The SPSS software (version 21.0; SPSS, Inc., Chicago, IL, United States) was used for data analysis. Descriptive statistics are given as means and standard deviations (SD) or frequencies (percentages). All variables were checked for normality before the analysis using the Kolmogorov–Smirnoff test. Differences between the means at kindergarten, 1st grade, and 5th grade were analyzed with one-way ANOVA. Mixed ANOVA was performed to analyze differences between the groups formed according to sports club participation. The chi-square test was used to analyze differences with categorical values. The Pearson correlation analysis was applied to find associations between estimated parameters. The effect size (ES) was calculated and considered to be small if it was less than 0.2, moderate if it was more than 0.5, or large if it was more than 0.8. Significance was set at *p* < 0.05.

## Results

The characteristics of children at 6.6 (kindergarten), 7.6 (1st grade), and 11.6 (5th grade) years are shown in Table 1. The BMI in 5th grade at school was higher than in kindergarten (*p* < 0.05). The ES of the difference in the BMI value was more pronounced in group 1 as the value of the BMI was highest in group 3 (Table 1). The body fat % in group 1 decreased (*p* < 0.05) from kindergarten to school, but in 5th grade, it was not different from the value in kindergarten (Table 1). At the same time, in groups 2 and 3, the body fat % in 5th grade was higher than the value in kindergarten (*p* < 0.05; ES = 0.13 and ES = 0.62, respectively) (Table 1). The FFMI increased significantly (*p* < 0.05) in all groups studied during the period from kindergarten to 5th grade, being most expressed in group 1 (ES = 1.77) (Table 1). The increase of the FFMI from kindergarten to 1st grade was also significant in all groups studied (Table 1). The 20-m shuttle run results expressing CRF improved from kindergarten to 1st grade and from 1st grade to 5th grade in all groups studied (*p* < 0.05), but the improvement was most expressed in group 1 (*p* < 0.05; ES = 0.81) (Table 1). The daily levels of MVPA and VPA did not differ in neither the group of children nor in kindergarten or school (Table 1).

**TABLE 1:** Body composition indices, 20-m shuttle run results, and physical activity of study participants in different groups according to age and organized sports participation.

Variable *n*; Boys/girls	Group 1: 48 (20/28) in kindergarten, 57 (25/32) in 1st grade, and 56 (27/29) in 5th grade	Group 2: 54 (26/28) in kindergarten, 25 (12/13) in 1st grade, and 62 (31/31) in 5th grade	Group 3: 110 (64/46) in kindergarten, 54 (33/21) in 1st grade, and 25 (14/11) in 5th grade
BMI (kg/m^2^)			
Kindergarten	15.9 ± 1.4	15.8 ± 1.5	16.2 ± 2
1st grade	16.1 ± 1.8	16.0 ± 1.9	16.6 ± 2.4**
5th grade	18.5 ± 2.6*; ^#^(ES = 0.5)	18.3 ± 3.4 ^#^(ES = 1.0)	20.7 ± 5.2 ^#^(ES = 1.1)
Body fat %			
Kindergarten	20.1 ± 4.4	20.4 ± 4.6	21.3 ± 4.5
1st grade	17.4 ± 4.8^#^	17.7 ± 6.0^#^	18.0 ± 5.0
5th grade	21.1 ± 6.3*(ES = 0.6)	21.3 ± 8.0*; **(ES = 0.1)	26.1 ± 9.9**(ES = 0.6)
FFMI (kg/m^2^)			
Kindergarten	12.6 ± 0.8	12.6 ± 1.0	12.3 ± 1.1
1st grade	13.2 ± 1.0^#^	13.1 ± 0.8^#^	13.5 ± 1.3^#^
5th grade	14.5 ± 1.2**(ES = 1.8)	14.2 ± 1.4**(ES = 1.3)	14.7 ± 1.6**(ES = 1.7)
20-m shuttle run (laps)			
Kindergarten	21.8 ± 9.8*(ES = 0.5)	21 ± 10.8*	17.0 ± 8.8
1st grade	24.3 ± 14.9^#^	25.4 ± 12.6^#^	22.1 ± 12.5^#^
5th grade	31.3 ± 13.5*; **(ES = 0.8; 0.8)	29.6 ± 16.4*; **(ES = 0.8)	20.7 ± 12**(ES = 0.6)
Daily MVPA (min)			
Kindergarten	68.4 ± 20.7	63.4 ± 16.7	71.2 ± 24.3
1st grade	74.8 ± 24.7	66.2 ± 21.1	74.5 ± 29.2
5th grade	55.6 ± 20.7	62.9 ± 24.7	63.4 ± 24.1
VPA (min/day)			
Kindergarten	20.6 ± 10.5	18.6 ± 7.2	21.7 ± 11.3
1st grade	25.0 ± 12.5	19.3 ± 9.3	24.8 ± 14.7
5th grade	18.4 ± 11.7	22.2 ± 14.5	22.6 ± 16.9

*p < 0.05 as compared to group 3. ^#^ *p* < 0.05 as compared to the results in kindergarten. **p < 0.05 as compared to the results in 1st grade.

Group 1—participation in organized sports in all stages of the study. Group 2—participation in organized sports in 5th grade or in 1st and 5th Grades. Group 3—never participated in organized sports.

BMI, body mass index; FFMI, fat-free mass index; MVPA, moderate-to-vigorous physical activity; VPA, vigorous physical activity; ES, effect size (Cohen’s D).

The comparisons of body composition indices, 20-m shuttle run results, and daily PA values of children at three time points according to the participation rate in organized sports are shown in Table 1. In addition, the BMI and body fat % of group 1 were different from those indices in group 3 (*p* < 0.05; ES = 0.53 and ES = 0.62, respectively) (Table 1). Differences in the body fat % can also be seen between groups 2 and 3 (*p* < 0.05) (Table 1). The FFMI did not differ in comparison according to the organized sports participation rate (Table 1). The results of the 20-m shuttle run of group 1 differed significantly from the results of group 3 kindergarten and 5th grade (*p* < 0.05; ES = 0.52 and ES = 0.83, respectively). The 20-m shuttle run results of group 2 also exceeded the results of group 3 (*p* < 0.05). The daily levels of MVPA and VPA did not differ significantly either in kindergarten or school in all groups studied (Table 1).

**FIGURE 1:**
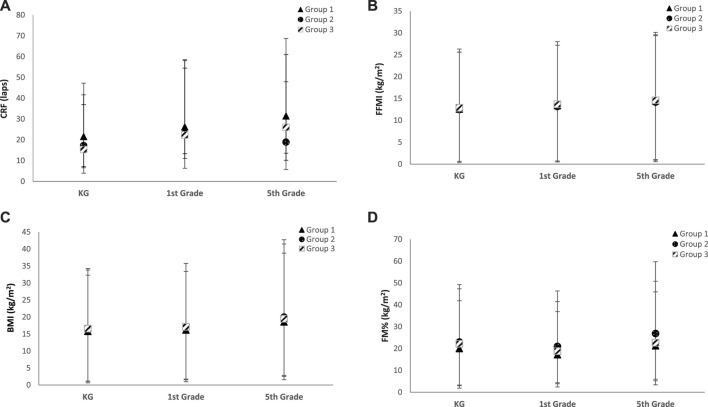
**(A)** Longitudinal changes in CRF in different groups according to organized sports participation. Estimated marginal means express the number of laps in the 20-m shuttle run. **(B)** Longitudinal changes in the BMI in different groups according to organized sports participation. Estimated marginal means express the value of the BMI. **(C)** Longitudinal changes in the FFMI in different groups according to organized sports participation. Estimated marginal means express the value of the FFMI. **(D)** Longitudinal changes in the body fat % in different groups according to organized sports participation. Estimated marginal means express the percentage of body fat. Groups 1, 2, and 3: group 1—participation in organized sports in all stages of the study; group 2—never participated in organized sports; and group 3—participation in organized sports in 5th grade or in 1st and 5th grades. CRF, cardiorespiratory fitness; MVPA, moderate-to-vigorous physical activity; VPA, vigorous physical activity; BMI, body mass index (kg/m^2^); FFMI, fat-free mass index (kg/m^2^).

Changes in CRF and body composition values are shown in Table 2 and Figures 1A–D using estimated marginal means to characterize the trends in measured indices more precisely.

**TABLE 2:** Estimated marginal means with confidence intervals of the 20-m shuttle run and body composition indices in different groups according to organized sports participation.

Variable	Group	Time	Estimated marginal mean	Std. error	95% confidence interval	
					Lower bound	Upper bound
20-m shuttle run (laps)	Group 1	Kindergarten	21.6	2.0	17.7	25.6
		1st grade	26.2	3.1	19.9	32.4
		5th grade	31.5	2.8	25.8	37.3
	Group 2	Kindergarten	17.3	3.5	10.2	24.3
		1st grade	23.0	5.5	12.0	34.1
		5th grade	18.9	5.0	8.7	29.0
	Group 3	Kindergarten	15.3	3.1	9.0	21.6
		1st grade	22.3	4.9	12.4	32.2
		5th grade	26	4.5	16.9	35.1
BMI (kg/m^2^)	Group 1	Kindergarten	15.8	0.3	15.2	16.5
		1st grade	16.2	0.5	15.2	17.2
		5th grade	18.6	0.8	17.0	20.2
	Group 2	Kindergarten	16.3	0.6	15.1	17.5
		1st grade	17.0	0.9	15.2	18.7
		5th grade	20.0	1.4	17.2	22.8
	Group 3	Kindergarten	16.6	0.5	15.6	17.7
		1st grade	17.1	0.8	15.6	18.7
		5th grade	19.5	1.2	17.0	22.0
FFMI	Group 1	Kindergarten	12.6	0.2	12.3	13.0
		1st grade	13.4	0.2	12.9	13.8
		5th grade	14.5	0.3	13.3	15.1
	Group 2	Kindergarten	12.5	0.3	11.9	13.1
		1st grade	13.2	0.4	12.5	14.0
		5th grade	14.2	0.5	13.1	15.2
	Group 3	Kindergarten	12.9	0.3	12.3	13.4
		1st grade	13.7	0.3	13.0	14.3
		5th grade	14.6	0.4	13.7	15.5
Body fat%	Group 1	Kindergarten	20.0	0.9	18.1	21.9
		1st grade	17.2	1.2	14.8	19.7
		5th grade	21.3	1.7	17.9	24.6
	Group 2	Kindergarten	23.0	1.6	19.7	26.3
		1st grade	21.0	2.2	16.7	25.4
		5th grade	26.9	2.9	20.9	32.8
	Group 3	Kindergarten	22.2	1.5	19.2	25.1
		1st grade	18.8	1.9	14.9	22.7
		5th grade	22.7	2.6	17.4	28.1

Group 1—participation in organized sports in all stages of the study. Group 2—participation in organized sports in 5th grade or in 1st and 5th rades. Group 3—never participated in organized sports.

BMI, body mass index; FFMI, fat-free mass index; body fat %, percentage of body fat.

The associations between 20-m shuttle run results, body composition indices, and PA in kindergarten and 5th grade in the groups according to organized sports participation are shown in Table 3. In the whole study sample, the 20-m shuttle run result expressing CRF was associated with the same measurement in kindergarten (*p* < 0.01) (Table 3). The BMI and body fat % were associated negatively with 20-m shuttle run results in both kindergarten and 5th grade (*p* < 0.01) (Table 3). Simultaneously, the association between daily MVPA and VPA levels and 20-m shuttle run results of the whole study sample was found only in kindergarten (*p* < 0.01). A positive association was revealed in group 1 between the 20-m shuttle run result of kindergarten and 5th grade (*p* < 0.01). The body fat% and 20-m shuttle run result of group 1 associated negatively in kindergarten (*p* < 0.01), but in 5th grade, the association was not significant (Table 3). At the same time, the MVPA level in kindergarten was positively associated with the 20-m shuttle run results in 5th grade (*p* < 0.01) (Table 3). In group 2, the 20-m shuttle run in kindergarten was associated with the same test result in 5th grade (*p* < 0.01) (Table 3). In group 3, the results of the 20-m shuttle run in kindergarten and 5th grade were more strongly associated than those of groups 1 and 2 (*r* = 0.736 vs. *r* = 0.461, and *r* = 0.513; *p* < 0.01) (Table 4). At the kindergarten level, no associations were found between body composition indices and 20-m shuttle run results, but there was a negative association between the body fat% and the 20 m shuttle run result in 5th grade (*p* < 0.01). The daily time spent in MVPA and VPA levels was associated with the 20-m shuttle run result of group 3 in kindergarten (*p* < 0.05) but no more in 5th grade (Table 3).

**TABLE 3:** Statistically significant associations between 20-m shuttle run results, body compositions indices, and physical activity in kindergarten and 5th grade in groups of study participants according to organized sports participation.

	Whole sample	Whole sample	Group 1	Group 1	Group 2	Group 2	Group 3	Group 3
Variable	20 m SR KG (r)	20 m SR 5.G (r)	20 m SR KG (r)	20 m SR 5.G (r)	20 m SR KG (r)	20 m SR 5.G (r)	20 m SR KG (r)	20 m SR 5.G (r)
20 m SR KG	-—	0.513**	—	0.461**	—	0.466**	—	0.736**
20 m SR 5.G	0.513**	—	0.461**	—	0.466**	—	0.736**	—
BMI KG	−0.203*^a^	—	—	—	−0.337**	—	—	—
BMI 5.G	−0.280**	−0.308**	—	—	−0.443**	−0.398**	—	—
BF% KG	−0.211**	−0.252**	-0.294*	—	—	—	—	—
BF% 5.G	−0.335**	−0.423**	—	—	−0.419**	−0.432**	—	−0.635**
MVPA KG	0.223**	—	—	0.346**	—	—	0.325**	—
VPA KG	0.200**	—	—	—	—	—	0.306**	—

r-Pearson’s correlation coefficient. *p < 0.05. **p < 0.01.

r 20 m Sr KG, 20 m shuttle run results in kindergarten; 20 m SR 5.G, 20 m shuttle run results in 5th Grade; BMI KG, body mass index in kindergarten; BMI 5.G, body mass index in 5th Grade; BF% KG, body fat percentage in kindergarten; BF% 5.G, body fat percentage in 5th Grade; MVPA KG, moderate-to-vigorous physical activity in kindergarten; VPA KG, vigorous physical activity in kindergarten.

Group 1—participation in organized sports in all stages of the study. Group 2—participation in organized sports in 5th grade or in 1st and 5th grades. Group 3—never participated in organized sports

**TABLE 4:** Percentage of 11.5-year-old children with healthy cardiorespiratory fitness according to participation in organized sports.

Participants	Group 1	Group 2	Group 3
Girls^a^	*n* = 17; 58%*^#^	*n* = 13; 41%*	*n* = 0; 0%
Boys^b^	*n* = 24; 88%*^#^	*n* = 12; 38%	*n* = 6; 42%
Whole sample	*n* = 56; 73%*^#^	*n* = 64; 39%*	*n* = 25; 24%

a 20 m shuttle run result ≥24 laps.

b 20 m shuttle run result ≥31 laps.

*p < 0.05 as compared to group 3. ^#^ *p* < 0.05 as compared to group 2. Group 1—participation in organized sports in all stages of study. Group 2—participation in organized sports in 5th Grade or in 1st and 5th Grades. Group 3—never participated in organized sports.

The percentage of 5^th^-grade children with healthy CRF is shown in Table 4. The percentage of children with healthy CRF in group 1 was almost threefold (73%) when compared with those in group 3 (24%) (Table 4). In group 3, no girls were on the age-appropriate CRF level according to their 20-m shuttle run result (Table 4).

## Discussion

The main purpose of the present three-wave study was to compare CRF and body composition indices of children during the transition from kindergarten to basic school according to their participation in organized sports. Three different groups based on participation in sports clubs were formed retrospectively after the third stage of the study. The attendance of sports clubs in all stages of the study was considered. The children were categorized as consistently, episodically, or never attended sports clubs in the duration of 5 years. The main finding of the study revealed that consistent attendance in organized sports in childhood and early preadolescence ensures higher CRF and healthier body composition compared with children who had no experience of organized sports. The trends of longitudinal changes in body composition were similar in all groups studied, but the children who participated in sports clubs during the whole study period showed significantly lower body fatness values in 5th grade, being 11.5 years old, than non-participants of sports clubs. No associations between body fatness indices and CRF were found in 5th grade among consistently trained children, contrary to their episodically or not trained peers. The proportion of 5th grade children demonstrating age-appropriate healthy CRF was almost threefold higher in the group of consistent sports training among both boys and girls than among non-members of sports clubs.

### Changes in Cardiorespiratory Fitness

Cardiorespiratory fitness is an informative measure of the body’s ability to perform PA and exercise, and it also provides an important indicator of health (Ortega et al., 2008). In children and adolescents, favorable associations have been reported connecting CRF and musculoskeletal fitness to cardiometabolic disease risk, adiposity, mental health, and cognition and musculoskeletal fitness to bone health (Ruiz et al., 2009; Smith et al., 2014). In the present study, CRF was assessed using a standardized 20-m shuttle run test which is reliable and practically feasible in kindergarten and school settings (Ruiz et al., 2010; Cadenas-Sanchez et al., 2016b; Tomkinson et al., 2019). The results of our study showed that CRF expressed as the result of the 20-m shuttle run test increased significantly over time in all groups investigated. The improvement of CRF was most considerable among children who had attended sports clubs from at least the last year of kindergarten to 5th grade in basic school. In 11.5 years, they had practiced sports for approximately 5 years or even more. As the awareness about the importance of PA has risen in Estonian society, it is very popular to organize extracurricular PA already in the early years, and many parents encourage their children to attend sports clubs when they are still in kindergarten. The most popular sports events practiced in kindergarten years have been gymnastics for girls and soccer for boys (Riso et al., 2019a). The children who had joined with organized training somehow later in primary school also significantly exceeded their peers in the 20 m shuttle run, who had never participated in sports clubs. The elementary school children were mostly engaged in athletics, swimming, and basketball in addition to gymnastics and soccer, which were the most practiced sports events in kindergarten. The common frequency of training in our study sample was 2–3 times per week. Entering school is a big event in the life of a child, and it has also been observed in previous studies that the transition from kindergarten to school is associated with a variety of negative changes. A decrease in the PA level has often been found after entry into elementary school (Lisowski et al., 2020). It must be mentioned that the level of PA did not decrease in 1st grade at school in the present study (Reisberg et al., 2020). The second stage of our study was carried out in 1st grade at school. All children demonstrated significant progress in CRF despite of their sports club membership or not. Although the training experience of this age was not remarkable yet, the CRF level of sports club members was higher than that of non-members already in kindergarten. It is interesting to note that the children not participating in organized sports progressed significantly in the 20-m shuttle run in 1st grade, but further development stopped and the results in 5th grade were below the results of 1st grade. The slower progress in endurance running among non-participants of sports clubs in basic school has also been previously observed (Roth et al., 2018). In 5th grade, at the third stage of the present study, the differences in CRF were more established between the study groups. These findings are in accordance with those of the study by Drenowatz et al. (2019), also confirming the importance of sustained sports practice in youth. Sports participation has also been shown to have a stronger relationship with CRF than non-organized leisure-time PA (Jaakkola et al., 2009). This may at least partially be associated with the energetic demands of sports club participation as habitual PA bursts may not be of sufficient volume and intensity to modify CRF (Campos et al., 2017; Santos et al., 2018). Accordingly, sports club participation has been related to greater total PA and a higher probability of meeting PA recommendations (Kokko et al., 2018; Lee et al., 2018). Particularly, recommendations for VPA have been more definitively achieved by sports club participants (Kokko et al., 2018; Riso et al., 2019b), and there appears to be a beneficial association of sports participation during childhood and adolescence with PA levels during adulthood (Azavedo et al., 2007).

Direct evidence has also emerged indicating that low CRF in adolescence is significantly associated with all-cause mortality later in life (Ortega et al., 2012; Högström et al., 2016). Education experts have considered that when children take part in sports and physical education, these physical activities contribute to their mental acuity and skills (Tomporowski et al., 2011). Previous studies have also revealed that more fit children achieved better results in cognitive tests (Riso et al., 2019b; Reisberg et al., 2021). The childhood CRF level has been stated as a predictor of present and future health status (Smith et al., 2014), but over the past decades, it has rapidly declined (Hardy et al., 2013). Accordingly, because of its popularity, sports club participation should be recommended as an effective strategy to reduce fatness and increase fitness in children (Zahner et al., 2006; Roth et al., 2018). The World Health Organization also promotes the use of existing settings based on the national situation and cultural habits for the prevention of overweight and obesity (Chaput et al., 2020).

### Associations Between Cardiorespiratory Fitness, Body Composition, and Physical Activity

The absence of associations between body composition indices and CRF in consistently trained 5th grade children allows us to conclude that physical fitness was not related to body fatness in this age group. On the other hand, 5th grade children who had never been members of sports clubs or had trained episodically showed a negative association between body fatness and CRF. This is in accordance with finding about the interaction between adiposity and CRF, suggesting that high levels of CRF may attenuate the adverse effects of being overweight or obese in children and youth, the so-called “fat but fit” phenotype (Eisenmann et al., 2005). In contrary to 5th grade children having several years of sports club experience, a negative association was found between body fat% and BMI with the 20-m shuttle run result in kindergarten children. Previous data from the same cohort (Riso et al., 2019b) in the baseline of the study and other investigations with preschool children (Ara et al., 2004; Zahner et al., 2009) have shown that overweight children achieved weaker results in the 20-m shuttle run test, although the participation rate in organized sports was equal with their normal-weight peers. Consequently, it could be recommended that sustained periods of training are beneficial for children, regardless of their body weight or fatness. Consistent physical efforts help improve physical fitness and attenuate the negative effects of fatness.

As CRF is a weak-to-strong predictor of cardiovascular disease risk, cancer, and mental health in children and youth (Ortega et al., 2008; Ruiz et al., 2011), achieving a healthy CRF level is of critical importance. Accordingly, CRF characterizes synergistic capabilities of several bodily systems and organs that are involved in the performance of PA and exercise, providing a strong and summative measure of health in children and youth (Ortega et al., 2008). The associations between PA, CRF, and various health outcomes are well-established in adults (Högström et al., 2014). Higher levels of PA and CRF are associated with lower levels of body fatness in children (Leppänen et al., 2018) and adolescents (Lätt et al., 2015) and have been stated to reduce the risks associated with obesity and other cardiovascular disease risk factors later in life (Högström et al., 2014).

### Compliance to the Reference Normative of Cardiorespiratory Fitness

Gender- and age-specific CRF reference normatives are used to classify the level of physical fitness in children and to monitor the fitness status of the population. Although physical fitness references have been reported in Spain and some other European countries, harmonized normative values applicable throughout Europe are needed for children and adolescents (Ortega et al., 2011). The 20-m shuttle run test is arguably the most popular field-based assessment and estimate of CRF in children and youth worldwide (Tomkinson and Olds, 2008). CRF also tracks moderately well from childhood to adulthood, indicating that the level of CRF in children provides an insight into present and future population health statuses (Ortega et al., 2013). The international normative 20-m shuttle run test values from more than 1.1 million children and youth representing 50 countries were used to compile the age-appropriate criterion-referenced standards for healthy CRF (Tomkinson et al., 2016). As 20-m shuttle run criteria have been calculated for children and youth from the age of 9 , we could evaluate the compliance of our participants in 5th grade only. The proportion of children demonstrating accordance to healthy CRF criteria was the highest in the consistently trained group, expectedly. Although the average 20-m shuttle run test result in episodically trained children was not significantly lower than in their consistently trained peers, the percentage of compliant children was almost twofold lower (38% vs. 73%). It seems that persistent participation in organized sports had a notable effect among boys, while 88% of them had a healthy CRF level. It could be considered a public health concern that 5th grade girls among the group of non-participants of sports clubs showed poor results in the 20-m shuttle run test, and none of them met the age-appropriate criterion of healthy CRF. This is a clear sign to local stakeholders to make sports activities for children and youth accessible for all demographic groups. Earlier results from the same study cohort have also shown that children from less educated families participate in organized sports less than children from more educated families, having probably better access to leisure time activities (Riso et al., 2019b).

The design of the three measurement points could be mentioned as the major strength of the present study, showing the importance of consistent participation in organized sports already in childhood and its associations with the development of CRF and body composition in the transition from kindergarten to basic school. The use of accelerometers to objectively measure PA and the application of the 20-m shuttle run test used both in PREFIT (Cadenaz-Sanchez et al., 2016a) and ALPHAFIT (Ortega et al., 2011) test batteries are reliable and practically feasible (Tomkinson et al., 2016) and could also be considered a strength of this study. The present study has also some limitations as body composition was measured indirectly using skinfold thicknesses. However, the assessment of body composition using skinfold thicknesses is a more sensitive marker than the BMI in the determination of body fat% in children (Cicek et al., 2014). Adjusting analysis has not been performed in the present study, so the results should be interpreted with caution. Also, the missing within-case longitudinal analysis and small sample size do not allow the generalization of the results to the whole population.

## Conclusion

The development of CRF and the percentage of children compliant with the age-appropriate CRF criterion were highest in children who trained consistently in sports clubs during the whole study period. No associations were found between body fatness indices and CRF in children who participated in sports clubs during the whole study period, contrary to children who joined sports clubs later or never. Consistent participation in organized sports from kindergarten to basic school is associated with lower body fatness in 11.5-year-old children.

However, the results of the present study should be not generalized to the whole population because of the missing adjustment of variables and the absence of a within-case longitudinal analysis. A more precise interpretation of data should be necessary for future studies.

## Data Availability

The raw data supporting the conclusion of this article will be made available by the authors, without undue reservation.
